# Detection of Hatching Information of Meat Duck Eggs Based on Deep Learning

**DOI:** 10.3390/ani15233400

**Published:** 2025-11-25

**Authors:** Jiawen Cai, Yongsheng Li, Yi Ji, Min Xu, Zhimin Liu, Yue Wang, Huixin Wang, Enze Duan, Lianfei Huo, Yunping Liu, Yonghong Zhang, Honghua Huan, Guofeng Han, Zongchun Bai

**Affiliations:** 1 College of Agricultural Engineering, Jiangsu University, Zhenjiang 212013, China; 2 Institute of Agricultural Facilities and Equipment, Jiangsu Academy of Agricultural Sciences, Jiangsu Province Facility Water Poultry Health Breeding Equipment Engineering Research Center, Key Laboratory of Protected Agriculture Engineering in the Middle and Lower Reaches of Yangtze River, Ministry of Agriculture and Rural Affairs, Nanjing 210014, China; 3 Remhart (Zhenjiang) Intelligent Technology Co., Ltd., Zhenjiang 212009, China; 4 Shuyang Zhongke Poultry Breeding Co., Ltd., Suqian 223625, China; 5 School of Automation, Nanjing University of Information Science and Technology, Nanjing 210044, China

**Keywords:** egg classification, deep learning, residual network, attention mechanism

## Abstract

Accurate and rapid identification of duck hatching status during early-to-middle incubation stages is critical for current duck egg incubation practices. To address the limitations of manual candling inspection, this study employed machine vision as an alternative to manual egg detection and proposed an improved residual neural network for assessing the developmental status of duck eggs. Compared with existing convolutional neural networks, the proposed model incorporates residual modules for skip connections, integrates CBAM attention modules for optimization, and utilizes advanced hyperparameter optimization algorithms, including the Adam optimizer. The model was evaluated ten times on the test set, and the optimized network showed an 8.62% improvement in accuracy with a 1.1% reduction in variance compared to baseline models. Experimental results on duck egg datasets revealed that the Improved ResNet34 model achieved an overall classification accuracy of 96.03%. These findings indicate that the Improved ResNet34 architecture exhibits superior classification performance for duck egg status assessment during early-mid incubation stages, with high recognition accuracy, robust generalization ability, and substantial potential for industrial applications.

## Simple Summary

To address the inefficiencies and errors of manual candling in duck egg incubation, this study developed an improved ResNet34 model integrated with a Convolutional Block Attention Module. The model, which employs machine vision for candling, achieved 96.03% classification accuracy in detecting unfertilized, live embryonated, and abnormal duck eggs during early-to-mid incubation. It showed faster convergence (within five epochs) and better stability than baseline models, demonstrating strong potential for industrial implementation in automated duck egg hatching processes.

## 1. Introduction

With the steady growth in national per capita disposable income and the pursuit of improved dietary nutrition, food consumption has become increasingly diversified. This has led to a growing demand for meat ducks, thereby expanding the scale of duck farming and hatching operations. Current duck egg hatching processes require candling in the early-to-mid incubation stage to identify and remove non-viable eggs [1]. Traditionally, this task relies on manual labor, which is time-consuming, labor-intensive, and prone to errors during extended working periods. Workers must perform high-intensity work within a limited timeframe, and prolonged work duration reduces accuracy, significantly impairing detection efficiency. Moreover, manual candling efficiency is relatively low: a single egg cart holds approximately 10,000 eggs, and removing all eggs from the incubator may disrupt subsequent hatching and increase the dead embryo rate [2]. Excluding the effects of fatigue, the efficiency of manual egg handling is approximately 5000 eggs per hour.

Although intelligent candling devices for duck eggs have been developed (primarily adapted from chicken egg hatching models) [3,4], they often fail to account for duck-specific physiological characteristics. As waterfowl, ducks have distinct requirements compared to chickens, including thicker yet more fragile eggshells, higher heat dissipation needs, and varied temperature, humidity, and incubation duration requirements [5,6]. Thus, developing a duck-specific candling classification method tailored to actual hatching conditions is urgently needed.

Scholars from China and abroad have conducted extensive and multidimensional research on candling methods for hatching eggs, yielding numerous scientific achievements. Previous scholars have attempted to use acoustics-based approaches [7]; however, this method relies on external environmental factors in practical application and lacks stability. Most studies have focused on histological analysis of embryos in hatching eggs during incubation but have failed to achieve non-destructive and rapid detection. Some teams have also explored spectroscopic approaches to improve accuracy, whether through hyperspectral transmission or near-infrared spectroscopy, yet such methods entail high equipment costs [8,9,10,11]. Consequently, scholars have shifted their focus to photoelectric sensors and machine vision [12,13,14,15,16,17]. Photoelectric sensors exhibit significant advantages in distinguishing fertile and unfertilized eggs, but existing hatcheries are not satisfied with distinguishing only these two categories [18]; practical demands require inclusion of both viable and dead embryos. Finally, through continuous breakthroughs in machine vision combined with emerging deep learning methods [19], scholars have achieved some progress. In addition, these technologies have also been applied to gender identification and have mostly yielded favorable results [20,21,22,23,24,25].

Existing hatching detection methods exhibit significant limitations for duck egg incubation. While histological analysis offers high accuracy, it is destructive and ill-suited for real-time incubation monitoring. Spectral technology, though effective, requires expensive equipment; furthermore, owing to duck eggs’ thicker shells than chicken eggs, light penetration is unstable, thereby compromising detection accuracy. Photoelectric sensors, despite their cost-effectiveness, fail to differentiate between live embryos and early dead embryos due to poor signal discrimination ability. Although machine vision has emerged as a promising alternative, most research has focused on chicken eggs, overlooking the unique challenges posed by duck eggs: rough shell surfaces induce greater noise interference, and uneven light transmittance attenuates embryonic features. This underscores the urgent need for customized deep learning models tailored to duck eggs to address the industrial demand for high-throughput, non-destructive detection.

Despite the rapid advancements of deep learning, its application in waterfowl hatchery systems remains understudied—particularly for ducks, as research on ducks lags behind chicken-centric studies. While chicken incubation models provide a foundational framework, they are ill-equipped to account for duck-specific biological traits (e.g., thicker eggshells, delayed embryonic development) and industry-specific workflows. Significantly, China’s meat duck hatcheries are increasingly embracing intelligent incubation systems, yet they encounter a critical bottleneck: the absence of reliable technology to accurately classify eggs at the 12-day embryonic stage—a pivotal timeframe for optimizing hatching efficiency and minimizing losses associated with unfertilized or abnormal eggs.

## 2. Materials and Methods

This study was performed according to the Research Committee of the Jiangsu Academy of Agricultural Sciences and was carried out in strict accordance with the Regulations for the Administration of Affairs Concerning Experimental Animals [Permit Number SYXK (Su) 2020-0024].

### 2.1. Experiment Design for Image Collection

The image collection site is located at Zhongke Poultry Co., Ltd. in Shuyang County, Suqian City, China. The hatching base belongs to one of the largest Cherry Valley parent duck breeding enterprises in northern Jiangsu, with an annual output of 120 million ducklings, providing an adequate sample size for experimental data collection. The experiment was conducted from November to December 2024. An enclosed dark box (300 × 300 × 300 mm) was built indoors. The black box used aluminum profiles to form the overall frame, with black plastic panels as the box walls and dense black foam as the light-absorbing pad. To more clearly visualize the internal characteristics of breeding eggs, a 20,000 lux light source (Fortislux flashlight, Yiwu Aitisi E-commerce Co., Ltd., Yiwu City, China) was placed near the upper-middle part of the eggshells. The imaging camera was positioned outside the dark box, 500 mm away from the eggs. The camera utilized a PCB module independently developed by the Jiangsu Academy of Agricultural Sciences, with a resolution of 3840 × 2160 pixels.

**Figure 1:**
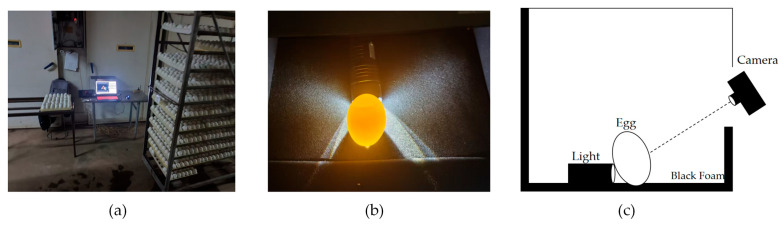
Collection method diagram: (**a**) Experimental site; (**b**) The captured footage; (**c**) Experimental framework diagram

The experimental site is shown in Figure 1, where Figure 1a,b are on-site photographs, and Figure 1c is a schematic diagram of the imaging angle. A total of 3664 Cherry Valley duck hatching eggs were selected for the experiment, including 1010 live embryonated eggs, 981 unfertilized eggs, and 1667 abnormal eggs. To avoid disrupting production and experiments, all selected duck eggs had already been incubated in the incubator for a specific number of days. The selected embryonic age corresponded to the timing when the hatchery conducted egg candling, typically at 12-day embryo age. Among these, unfertilized eggs and abnormal eggs were photographed first, both of which were manually selected. Live embryonated eggs, on the other hand, were duck eggs of the same embryo age and from the same egg cart, which continued to be incubated in the incubator after manual candling. By the time of photography, live embryonated eggs had been placed in the egg cart for a period after selection. During the experiment, measures were taken to ensure that live embryonated eggs were not left outside the incubator for more than one hour, and incubation temperature was maintained to minimize adverse impacts.

**Figure 2:**
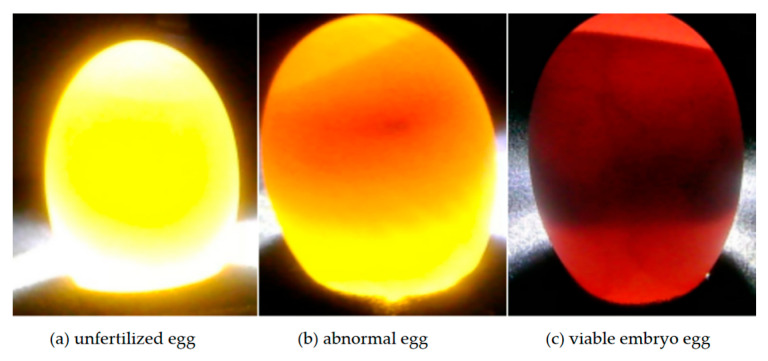
Egg classification.

Under a stable experimental environment, with the light source transilluminating the interior of the breeding eggs, the imaging module (3840 × 2160 pixels) was used to sequentially capture the internal images of each breeding egg, as shown in Figure 2. In Figure 2a, the unfertilized egg appears pale amber-yellow overall, with no foreign objects observed inside when transilluminated. Figure 2b shows an abnormal egg, which is tan-brown with prominent brown regions and may have distinct black spots in the middle. Figure 2c depicts a live embryonated egg, which is dark red overall, with distinct black blood vessels visible in some areas [26].

These observations reveal distinct differences in candling characteristics between duck eggs and chicken eggs. Excluding variations in shell morphology, duck eggs exhibit greater volume and more dispersed shadow regions, causing live embryos to appear dark red when candled, whereas chicken embryos appear light yellow under the same conditions. Consequently, the contrast between duck embryos and duck yolks differs significantly from that between chicken embryos and chicken yolks. While such differences would initially facilitate classification, the thicker and rougher shells of duck eggs heighten resistance to light transmission, resulting in uneven light distribution during candling. This, in turn, renders traditional color-threshold-based segmentation algorithms unstable, necessitating specialized preprocessing steps.

Furthermore, we conducted supplementary experiments using monochromatic light sources (red, green, and blue). Color filtering was implemented by attaching different color filters to the light source, which minimized light intensity loss and thereby maintained consistent intensity across experimental groups. Our results demonstrated that monochromatic light failed to provide sufficient visual information for embryo observation; moreover, the penetration efficiency of most monochromatic lights was insufficient to visualize the internal contents. Under controlled experimental conditions, white light with higher intensity yielded a superior visualization effect. Notably, this conclusion remains valid in the absence of specific constraints limiting increases in white light intensity.

### 2.2. Preprocessing of Duck Egg Image Data

To obtain more accurate image data with more abundant retained details, and considering the unique characteristics of duck eggs relative to chicken eggs, preprocessing of the acquired images was necessary. During the image capture process, the baffle of the light-tight box exhibited reflections under intense illumination. Additionally, the camera module was positioned 500 mm away from the duck eggs, resulting in the duck eggs occupying a relatively small area in the entire photograph. The full image resolution is 3840 × 2160 pixels, while algorithmic processing typically requires images of 244 × 244 pixels. Brute-force scaling would result in excessive loss of duck egg information; therefore, appropriate cropping was necessary to preserve more effective classification-relevant information.

**Figure 3:**
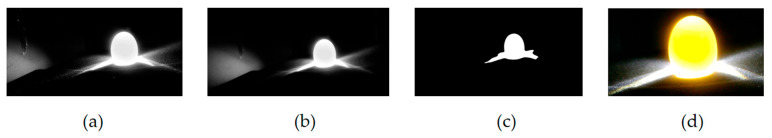
Preprocessing steps for duck egg image data: (**a**) Grayscale image; (**b**) Filtered image; (**c**) Threshold segmentation contour; (**d**) Processed image.

In image cropping, the image was first converted to grayscale, and a filter was applied to smooth the image content, as shown in Figure 3a. To preserve signal edges during filtering and prevent blurring, median filtering was employed, as shown in Figure 3b. Median filtering replaces the value of a point in a digital image or sequence with the median value of its neighbors’ values, enabling surrounding pixels to approximate true values and thereby eliminating isolated noise points. After filtering, it was observed that reflections were concentrated in a single area, opposite to the duck egg region. To identify the duck egg region, a combined method of Otsu threshold segmentation and manual offset was employed. Otsu is an adaptive image binarization method based on the maximum between-class variance principle. It finds the optimal threshold by iterating over grayscale levels, maximizing the inter-class variance between the foreground and background after segmentation, thus achieving efficient and automated image segmentation, as shown in Figure 3c. The core formula of the Otsu method is the between-class variance:

(1) $$\sigma_{B}^{2}\left(T\right)=\omega_{0}\left(T\right)\omega_{1}\left(T\right){\left[\mu_{0}\left(T\right)-\mu_{1}\left(T\right)\right]}^{2}$$

where $\omega_{0}\left(T\right)$ and $\omega_{1}\left(T\right)$ represent the proportions of foreground and background pixels after threshold *T* segmentation, respectively; $\mu_{0}\left(T\right)$ and $\mu_{1}\left(T\right)$ denote the average grayscale values of the two pixel classes.

After automatic threshold segmentation, the duck egg and reflection regions could be classified as the foreground, while the remaining areas were designated as the background. However, to accurately isolate the duck egg region, manual offset adjustment was still required. To ensure full penetration of the eggshell during egg imaging, a light source with an intensity of 20,000 lux was selected. After grayscale processing, the resulting images revealed that the grayscale values of light leakage from unfertilized eggs and eggshells ranged between 254 and 255 (with 255 being the maximum gray value), which caused the automated segmentation threshold to shift toward a mid-range value. However, after grayscale processing, the grayscale values of reflections on the dark-box wall should ideally fall within the range of 180–200. Directly setting an excessively high threshold was also inappropriate, as the grayscale values in the vicinity of live embryos were lower than those near unfertilized eggs. Through experimental testing, manually adjusting the offset by +100 was determined to be the optimal parameter, effectively filtering out the wall reflection areas of unfertilized eggs while simultaneously excluding the grayscale values near live embryos.

Additionally, a detection function was integrated into this processing workflow: after minimizing reflection areas to the greatest extent, the largest detection region was selected for cropping to ensure complete capture of the duck egg region. The cropping area was defined as 1.1 times the bounding box of the detection region to prevent truncation of the duck egg. The final result is presented in Figure 3d.

### 2.3. Establishment of Classification Model

**Figure 4:**
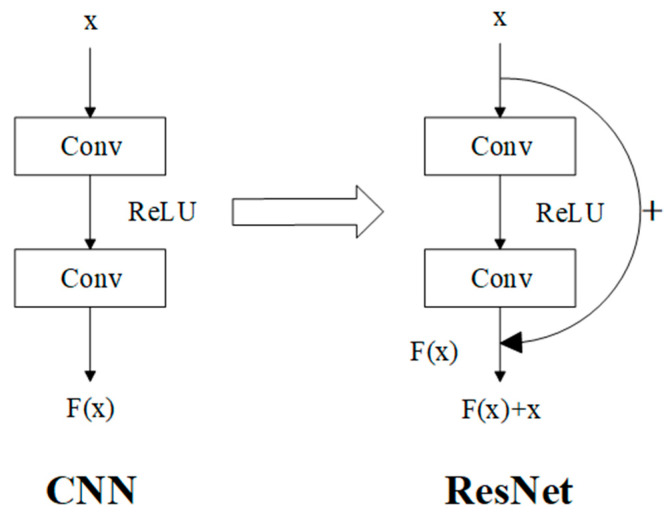
Basic residual block. Input *x*: image pixel matrix; arrow: calculation steps; +: sum of residuals.

Compared with Recurrent Neural Networks (RNNs) and Deep Neural Networks (DNNs), Convolutional Neural Networks (CNNs) demonstrate superior applicability to image data than the former and faster processing speeds than the latter. In the domain of image processing, CNNs exhibit broader suitability and a more extensive array of advanced algorithms. Residual Neural Network (ResNet) represents an advanced variant of convolutional neural networks [27], which introduces shortcut connections into the fundamental CNN architecture to mitigate the problem of model degradation in deep networks. Theoretically, a basic residual block is constructed by adding the original input to the convolutional layer—composed of convolutional operations and activation functions—within the network (Figure 4).

In the diagram, Conv stands for convolution; ReLU represents the activation function, while *x* and F(x) correspond to the input and output, respectively.

After improvement, the network does not encounter degradation issues even if it fails to learn anything during a single learning iteration. The specific residual block formula is as follows:

(2) $$y=F\left(x,\left\{W_{i}\right\}\right)+W_{s}x$$

In Equation (2), *y* represents the input and output of the residual layer, and formula $F\left(x,\left\{W_{i}\right\}\right)$ denotes the residual mapping to be applied. $W_{s}$ is a linear transformation used to match the dimensions of input and output for connection. In ResNet50 and ResNet101 architectures, dimensional mismatch may occur between the residual mapping and the input *x*, where a linear transformation is required for dimensional alignment. In this study, to meet practical production requirements, complex models were unnecessary, and ResNet34 was sufficient. The comparison of training effects among ResNet models with different depths is shown in Table 1 below:

All data in the table were derived from training on this study’s dataset. It should be noted that the accuracy in the table refers to the highest training accuracy and is not applicable as a final evaluation metric. It was only used as a criterion for algorithm selection. Based on the data in Table 1, ResNet50 and ResNet101 were discarded because their accuracy and training times failed to meet practical production requirements. ResNet18, although its highest accuracy differed from ResNet34 by only 1.6%, was also discarded due to excessive fluctuations during training and the need for numerous subsequent improvements. Therefore, this study adopted ResNet34 as the main algorithm for optimization.

### 2.4. Integration with Attention Mechanism

As mentioned earlier, compared to chicken eggs, duck eggs have thicker shells and uneven light transmission, which can cause the embryonic features in the image to appear less distinct. To address this issue, attention mechanisms need to be introduced to improve the algorithm.

Attention Mechanism is a technique in deep learning that mimics human visual and cognitive attention. By dynamically assigning different weights, it enables models to focus on key parts when processing information [28]. It is widely applied in fields such as Natural Language Processing (NLP) and Computer Vision (CV), significantly enhancing the model’s ability to handle long sequences and complex data. Recently, the attention mechanism has been widely utilized to improve the performance of convolutional neural networks. It helps neural networks focus on feature regions of images and reduce interference from redundant information.

Convolutional Block Attention Module (CBAM) achieves feature recalibration through cascaded channel and spatial attention [29]. Among these, the Channel Attention Module captures inter-channel dependencies through bidirectional aggregation in the spatial dimension:

(3) $$M_{C}\left(F\right)=\sigma \left(MLP\left(AvgPool\left(F\right)\right)+MLP\left(MaxPool\left(F\right)\right)\right)$$

In Equation (3), F denoted the input feature map; $AvgPool\left(F\right)\in R^{C}$ represented the spatial average pooling feature vector; $MaxPool\left(F\right)\in R^{C}$ was the spatial max pooling feature vector; MLP was a multi-layer perceptron with shared weights; and the Spatial Attention Module generated a spatial weight map through pooling operations in the channel dimension:

(4) $$F^{\prime }=M_{C}\left(F\right)⊗F$$

(5) $$M_{S}\left(F^{\prime }\right)=\sigma \left(f^{7\times 7}\left(\left[AvgPool\left(F^{\prime }\right);MaxPool\left(F^{\prime }\right)\right]\right)\right)$$

In Equation (4), $F^{\prime }$ was obtained by multiplying the channel attention weights above with the original feature map, where the multiplication was element-wise multiplication. In Equation (5), $AvgPool\left(F^{\prime }\right)\in R^{1\times H\times W}$ was the channel average pooling feature vector, $MaxPool\left(F^{\prime }\right)\in R^{1\times H\times W}$ was the channel max pooling feature vector, and $f^{7\times 7}$ was a 7 × 7 convolutional kernel. Finally, similar operations are repeated:

(6) $$F^{\prime \prime }=M_{S}\left(F^{\prime }\right)⊗F^{\prime }$$

**Figure 5:**
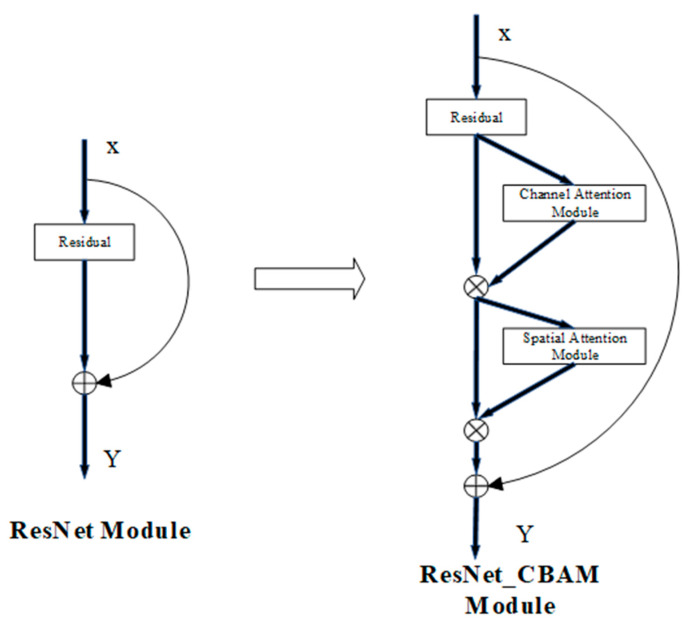
The fusion of CBAM and ResNet. Input *x*: image pixel matrix; *Y*: processed output features; arrow: calculation steps; +: Element-wise Addition; ×: Element-wise Multiplication.

In this way, the feature map $F^{\prime \prime }$, processed by CBAM attention, can be obtained. Taking the ResNet network as an example, as shown in Figure 5 below, *X* is the input, *Y* is the output, and the CBAM was directly inserted after the convolution module.

The unique channel-spatial dual attention mechanism of the CBAM effectively addresses the distinct physical characteristics of duck eggs. Owing to their thick outer shells, duck eggs exhibit poor and uneven light transmittance, which leads to weaker embryonic features in embryos under illumination. The CBAM first allocates attention to key embryonic features via channel attention while suppressing eggshell-related noise. Furthermore, through spatial attention, it mitigates illumination noise induced by variations in eggshell curvature and local thickness during duck egg embryo imaging. As a result, this ResNet-CBAM fusion algorithm inherits ResNet’s key strengths in achieving a balance between efficiency and performance, making it suitable for industrial-scale application in automated duck egg hatching processes.

### 2.5. Experimental Process and Evaluation Method

After data collection and image preprocessing as described above, the resulting dataset was split into training, validation, and test sets in a 6:2:2 ratio by sample count. There were three training targets: unfertilized eggs, abnormal eggs, and live embryonated eggs [30]. The classification criteria were established based on the operational requirements of the hatchery. Among these categories, unfertilized eggs are relatively straightforward to identify, with a critical distinction between live embryonated eggs and abnormal eggs requiring clarification. Abnormal eggs encompassed two subcategories: dead eggs and yolk-dispersed eggs. Dead eggs were characterized by the presence of embryos that have ceased development (i.e., non-viable embryos). Specifically, early-stage dead embryos presented as small, deep red or black foci within the yellow yolk in candling images, whereas late-stage dead embryos exhibited a distinct blackened appearance. In contrast, yolk-dispersed eggs exhibited a uniform yellow coloration, lacking both the pale coloration of infertile eggs and the presence of viable embryos.

Before training, the images underwent flipping, center cropping, normalization, dimensionality reduction, and other data augmentation and preprocessing processes to adjust them to standard specifications suitable for training, and training was conducted on the training set. After comparing models of various depths, the selected training model used 34 layers. In addition to the CBAM mentioned earlier, comparisons of fusion algorithms with classic attention mechanisms such as Coordinate Attention (CA) and Squeeze-and-Excitation Networks (SE) were also included.

**Figure 6:**
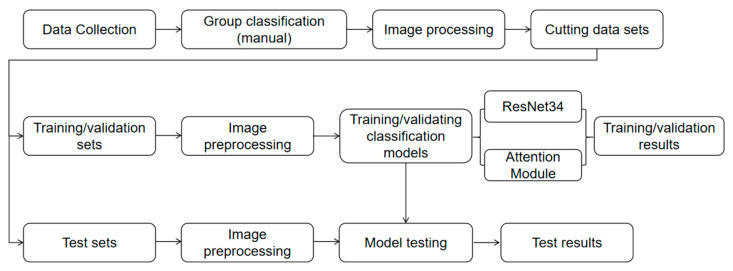
Overall flow chart of the experiment.

This experimental workflow was built on the PyTorch 2.3.1 deep learning framework under the Windows 10 operating system and was implemented with the Python 3.9.21 programming language. To align with practical production requirements and reduce hardware demands, experiments were conducted exclusively on an Intel Core i5-12600kf processor. In the model parameter configuration, given that the sample size was approximately 3000, 50 training epochs were selected to mitigate overfitting, and a batch size of 16 was chosen to reduce overfitting associated with large batch sizes. Additionally, the Adam optimizer, which was stable and fast-converging, was employed; it enabled automatic adjustment of the learning rate based on training dynamics, thus the default learning rate of 0.001 was adopted. Since the task is a classification task, cross-entropy loss was selected as the loss function. The specific experimental process is shown in the following Figure 6:

For the final practical application, the model evaluation metrics not only rely on classic model evaluation indicators such as accuracy but also include specific criteria conducive to practical use, such as convergence speed and model stability.

## 3. Results

**Figure 7:**
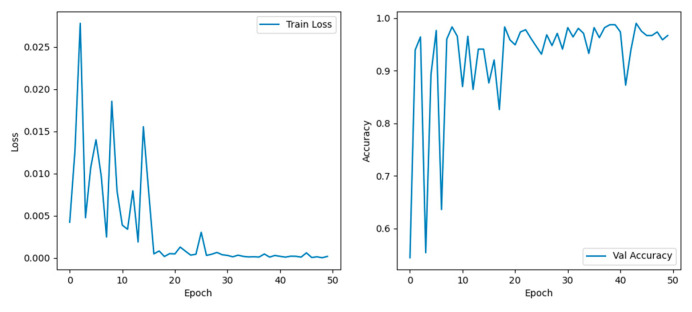
Training loss and accuracy variation graph in ResNet34.

The dataset, consisting of 3664 images across the three predefined categories, was split into training, validation, and test sets in a 6:2:2 ratio by sample count. Training was first conducted using 2202 images from the training subset with the ResNet34 network, and the training results are illustrated in Figure 7 below.

**Figure 8:**
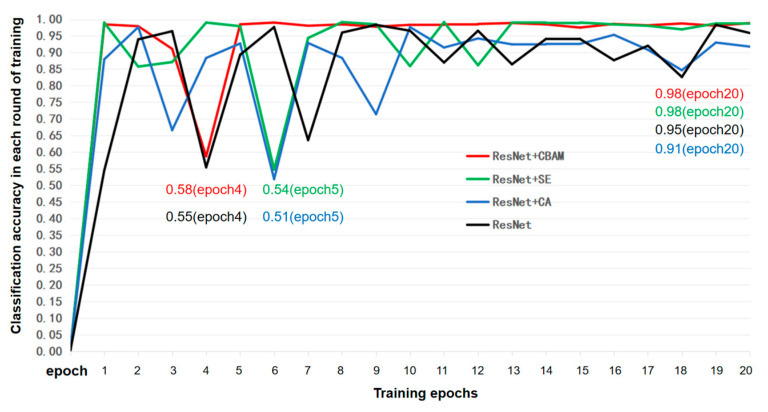
Comparison of classification accuracy with training epochs after the fusion of ResNet34 with SE, CA, and CBAM attention mechanisms.

In Figure 7, the left graph shows the changes in training loss as a function of training epochs, and the right graph shows the change in classification accuracy as a function of training rounds. It was clear that within the first 20 training rounds, both training loss and classification accuracy fluctuated significantly, then gradually stabilized thereafter. Regarding the accuracy changes within the first 20 rounds: accuracy fluctuated continuously before the 10th round; after the 10th round, it only exhibited a significant fluctuation of nearly 10% from the 12th to the 19th round, remaining relatively stable at other times. Meanwhile, the highest training loss only approached 3%. Overall, the classification accuracy reached a maximum of 99% and typically hovered around 95%. These results indicate that the classification task was relatively straightforward, thus obviating the need for excessively complex algorithms—which would otherwise increase computational workload and compromise efficiency. What we needed was to enable the algorithm to converge quickly and reduce random fluctuations. Therefore, the attention mechanism was added to accelerate convergence. In this study, SE, CA, and CBAM were selected as the modules to be added, and the specific training effects are shown in the following Figure 8:

To investigate the convergence behavior and stability of the model, the first 20 training epochs of the ResNet34-based algorithm were selected for analysis, as presented in Figure 8. The results indicated that the integration of the CA module failed to significantly enhance the convergence speed of the original ResNet34 algorithm; instead, this integration exhibited only a marginal advantage in stability after the 10th epoch. Similar to the CA module, the SE module also failed to remarkably improve convergence speed; however, it markedly enhanced classification accuracy after convergence stabilization. In contrast, the CBAM displayed transient fluctuations at the 4th epoch, which were rapidly recovered, thereafter maintaining stable performance at a consistently high accuracy level. To facilitate a more intuitive and precise comparison of the performance differences in convergence and stability among the three modules, four quantitative metrics—the average accuracy, overall variance, first 10-epoch variance, and the last 10-epoch variance—were calculated and summarized in Table 2:

In Table 2, in terms of average accuracy, CA was close to the original ResNet34 algorithm, while CBAM and SE ranked first and second, respectively. Variance represents the degree of data dispersion; to a certain extent, a smaller variance indicates less fluctuation. Whether it is the overall variance, variance of the first ten epochs, or the variance of the last ten rounds, the order of variance magnitude was CBAM > SE > CA > original model (without attention mechanism). These all confirm the content presented in the previous figures, and CBAM was the optimal result [31].

**Figure 9:**
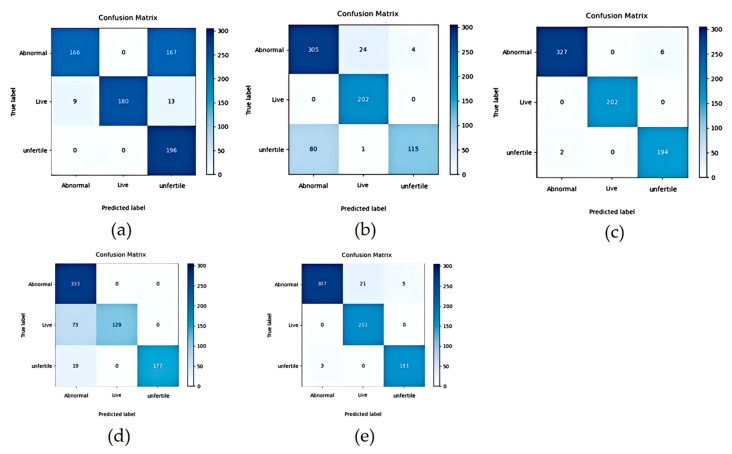
Confusion matrix of different models: (**a**) CNN, (**b**) AlexNet, (**c**) EfficientNet, (**d**) ResNet34, and (**e**) Improved ResNet34.

After training, various lightweight models were compared on the test set, including AlexNet, Shallow CNN, ResNet34, EfficientNet, and the improved ResNet34. To ensure result reliability, 10 rounds of testing were conducted. The models’ performance was evaluated using six indicators: accuracy, precision, recall, F1 score, average variance, and training time per epoch. The specific values are presented in Table 3 below:

Table 3 indicates that the time unit per testing epoch was seconds. Beyond specific time metrics, as research progresses, image classification algorithms have indeed achieved significant advancements. Among them, the currently prevalent EfficientNet algorithm demonstrated the optimal classification performance, with an accuracy of 98.91%, a precision of 98.8%, a recall of 99.06%, a F1 score of 98.92%, and an extremely low average variance of 0.006. However, during training, EfficientNet’s training time exceeded twice that of ResNet; this inefficiency was partially reflected in its testing phase: its testing time was longer than that of other compared models, potentially hindering independent optimization for incubator applications. The confusion matrices of these models were also shown in Figure 9 below:

The improved ResNet34-based model proposed in this study ranked second across the other evaluation indicators without significantly prolonging testing time, though substantial room for further improvement remains. Notably, these achievements were constrained by the fact that the images used in this study were captured under controlled dark-box experimental conditions, rather than complex real-world environments. Compared to the baseline ResNet34 model, the improved model exhibited an 8.62% increase in accuracy and a 0.011 reduction in variance.

## 4. Conclusions

This study employed image classification technology and deep learning to acquire images of 1010 live embryonated eggs, 981 unfertilized eggs, and 1667 abnormal eggs under light transmission conditions within a dark box. Following image preprocessing, the ResNet34 algorithm, suitable for industrial production, was adopted for deep learning. To enhance training performance, various attention mechanism modules (e.g., SE, CA, and CBAM) were incorporated for comparative analysis, and the key findings and implications are summarized as follows:

From an industrial perspective, duck egg enterprises emerged relatively late, and their incubation processes have been improved based on existing chicken egg incubation protocols. Notably, duck eggs and chicken eggs exhibit significant biological and physiological differences, which warrant further investigation. The improved ResNet34 algorithm proposed in this study attained an overall accuracy of approximately 96% and exhibited satisfactory stability. During testing, 731 images were processed within 25 s, corresponding to a theoretical throughput of 80,000 eggs per hour, meeting the requirements for industrial application. Nevertheless, several limitations should be noted. To achieve precise positioning of the light source in the experiment, factories are required to install light sources within the trachea and suction cup components and use the suction cup to closely adhere to the eggs for positioning. The ultimate recognition speed may be constrained by the speed of the assembly line and the movement of the suction cup. Additionally, different incubators may exhibit varying classification criteria, which necessitates further refinement of the model.

Furthermore, automation has been implemented in egg candling processes at some large-scale hatcheries, where machine vision combined with image algorithms exhibits significantly higher accuracy compared to alternative methods such as photoelectric detection. In contrast, many small-scale hatcheries still rely on manual candling. Intelligent candling systems outperform manual labor in both efficiency and stability, thereby reducing losses incurred during the candling process at hatcheries. This efficiency gain allows for more funds to be allocated to other incubation stages, collectively lowering the dead embryo rate and elimination rate across multiple stages of incubation. Consequently, intelligent candling holds great significance for large-scale breeding and improving animal welfare.

It should be noted that the eggs used in this experiment were collected from a single hatchery and limited to Cherry Valley meat ducks, introducing limitations regarding generalizability to other hatcheries and duck breeds. For instance, hatcheries often require more granular classification of live embryonated eggs and abnormal eggs, a scenario commonly observed in traditional hatchery operations. Additionally, the time required to test eggs may deviate from the 12-day incubation period, as such timing exhibits variability across multiple hatcheries. Although the algorithm model possesses a certain degree of generalization ability and operates on consistent underlying principles, adjustments are still required to adapt to practical conditions. Similarly, advanced instruments currently under development in the market are not fixed; instead, they require hatcheries to perform adjustments based on real-world scenarios.

The continuous expansion of breeding scale in the entire meat duck industry and the advancement of intelligentization are ongoing processes, among which intelligent incubation stands as an indispensable component. Currently, it is a critical period for promoting the adoption of intelligent egg candling in most enterprises, and this study aims to provide novel insights for the development of intelligent egg candling technology and resolve some problems.

## Figures and Tables

**Table 1:** Comparison of ResNet training at different depths.

ResNet Depth	Highest Precision	Training Time per Round (s)
18	96.7%	180
34	98.3%	320
50	97.7%	520
101	93.9%	900

**Table 2:** Comparison of ResNet34 and various attention fusion algorithms.

Algorithm	Average Accuracy	Overall Standard Deviation	Overall Variance	Variance in the First Ten Epochs	Variance in the Last Ten Epochs
ResNet34	0.878386	0.134118	0.017988	0.030856	0.002451
ResNet34 + CA	0.877360	0.111773	0.012493	0.020759	0.000735
ResNet34 + SE	0.937620	0.102252	0.010456	0.016896	0.001426
ResNet34 + CBAM	0.960597	0.087279	0.007618	0.014060	0.000016

**Table 3:** Comparison of results of various algorithm models on the test set.

	CNN	AlexNet	ResNet34	EfficientNet	Improved ResNet34
Accuracy	74.15%	85.09%	87.41%	98.91%	96.03%
Precision	82.33%	88.28%	92.78%	98.80%	95.70%
Recall	79.65%	83.42%	84.72%	99.06%	96.89%
F1	76.04%	84.05%	86.9%	98.92%	96.17%
Mean variance	0.029	0.012	0.018	0.006	0.007
Time Per round (s)	20	22	23	37	25

## Data Availability

Data are contained within the article.

## Abbreviations

The following abbreviations are used in this manuscript:

ResNet	Residual Neural Network
CBAM	Convolutional Block Attention Module
RNN	Recurrent Neural Network
DNN	Deep Neural Network
CNN	Convolutional Neural Network
CA	Coordinate Attention
SE	Squeeze-and-Excitation Network
